# Individual patient data meta-analysis of beta-blockers in heart failure: rationale and design

**DOI:** 10.1186/2046-4053-2-7

**Published:** 2013-01-18

**Authors:** Dipak Kotecha, Luis Manzano, Douglas G Altman, Henry Krum, Guliz Erdem, Nicola Williams, Marcus D Flather

**Affiliations:** 1 Clinical Trials and Evaluation Unit, Royal Brompton Hospital, Sydney Street, London, SW3 6NP, UK; 2 Monash Centre of Cardiovascular, Research and Education in Therapeutics, Monash University, Melbourne, VIC, Australia; 3 Department of Medicine, Universidad de Alcala, Hospital Universitario Ramon y Cajal, Madrid, Spain; 4 Centre for Statistics in Medicine, University of Oxford, Oxford, UK; 5 Norwich Medical School, University of East Anglia, Norwich, UK

**Keywords:** Beta-blockers, Heart failure, Meta-analysis, Design paper

## Abstract

**Abstract:**

The Beta-Blockers in Heart Failure Collaborative Group (BB-HF) was formed to obtain and analyze individual patient data from the major randomized controlled trials of beta-blockers in heart failure. Even though beta-blockers are an established treatment for heart failure, uptake is still sub-optimal. Further, the balance of efficacy and safety remains uncertain for common groups including older persons, women, those with impaired renal function and diabetes. Our aim is to provide clinicians with a thorough and definitive evidence-based assessment of these agents. We have identified 11 large randomized trials of beta-blockers versus placebo in heart failure and plan to meta-analyze the data on an individual patient level. In total, these trials have enrolled 18,630 patients. Uniquely, the BB-HF group has secured access to the individual data for all of these trials, with the participation of key investigators and pharmaceutical companies.

Our principal objectives include deriving an overall estimate of efficacy for all-cause mortality and cardiovascular hospitalization. Importantly, we propose a statistically-robust sub-group assessment according to age, gender, diabetes and other key factors; analyses which are only achievable using an individual patient data meta-analysis. Further, we aim to provide an assessment of economic benefit and develop a risk model for the prognosis of patients with chronic heart failure.

This paper outlines inclusion criteria, search strategies, outcome measures and planned statistical analyses.

**Trial registration:**

Clinical trial registration information: http://clinicaltrials.gov/ct2/show/NCT00832442

## Background

Heart failure (HF) is a major public health problem with both incidence and prevalence rising rapidly along with associated healthcare costs, estimated to be $39.2 billion in the United States and £625 million per year in the UK [1,2]. HF accounts for around 5% of all hospital admissions and re-admission rates approach 50% over the following 12 months. Prior to the 1990s, beta-blockers were considered to be contraindicated in HF. With an increased understanding of the pathophysiology of HF, the hypothesis developed that beta-blockers may alleviate inappropriate sympathetic drive, reduce heart rate and allow better cardiac filling. A series of small mechanistic studies followed by larger randomized trials have now established beta-blockers as a key evidence-based treatment to reduce mortality and morbidity alongside angiotensin converting enzyme (ACE) inhibitors and aldosterone antagonists. Current European and American guidelines give a class I recommendation for the use of beta-blockers in patients with symptomatic systolic HF [3,4].

However, survey data have confirmed that the uptake of beta-blockers in HF patients is still sub-optimal. Although the percentage of eligible patients prescribed beta-blockers increased between the first and second Euro Heart Failure surveys, a substantial number of patients remain untreated or receive sub-maximal therapy [5,6]. Paradoxically, those at the greatest risk of death are less likely to receive evidence-based therapy after a HF hospitalization [7]. The reasons for this are multi-factorial and include a long entrenched belief that starting beta-blockers in HF may make symptoms worse or that beta-blockers should only be commenced in specialized clinics. There has also been concern that the evidence-base is not representative of broader clinical practice and that common patient groups, including older persons, those with impaired renal function and diabetes may not benefit.

Although a number of sub-group and meta-analyses based on published data have been conducted [8,9], these can only address reported outcomes and are limited statistically. Only an individual patient data (IPD) meta-analysis is able to explore the effects of treatment on important secondary outcomes such as sudden death, NYHA class or ejection fraction and allow reliable pooled sub-group analyses.

## Methods

### The Beta-blockers in Heart Failure Collaborative Group (BB-HF)

The BB-HF group is a collaborative, multinational effort to combine individual data from the major randomized controlled trials (RCTs) investigating the use of beta-blockers in chronic HF. The group consists of the leading investigators of these trials and international experts, with the support of the four pharmaceutical companies that have marketed beta-blockers in HF (AstraZeneca, GlaxoSmithKline, Merck Serono and Menarini). A full list of collaborators is presented in Appendix A. Two meetings of the collaborative group in November 2008 and August 2010 were used to define our objectives, establish inclusion criteria and develop the primary and secondary objectives. A standardized data request form was generated to obtain IPD from each eligible trial (see Additional file 1)

At the time of this publication, individual data on 15,922 participants (representing 10 of the 11 trials) have been received by the coordinating center, the Clinical Trials and Evaluation Unit, Royal Brompton & Harefield NHS Trust/Imperial College London.

### Objectives

The aims and objectives of the BB-HF individual patient data meta-analysis include:

a) Provide a definitive estimate of the overall treatment effect of beta-blockers in HF on key outcomes including all-cause mortality and hospitalization.

b) Analyze the influence of important pre-randomization patient characteristics on the clinical effects of beta-blockers, including age, diabetes, gender, ejection fraction, renal function, atrial fibrillation and the etiology of HF.

c) Pool the adverse event and discontinuation data to assess the safety of beta-blockers in HF patients, particularly the rate of bradycardia and hypotension.

d) Explore the relationship between the effects of beta blockers and key baseline measurements including heart rate, blood pressure and weight.

e) Perform an exploratory analysis to assess important post-randomization variables, such as change in heart rate, achieved heart rate, change in blood pressure, achieved dose and the prescription of other concomitant therapies, such as ACE inhibitors, angiotensin receptor blockers (ARB), aldosterone antagonists, diuretics and digoxin.

f) Describe in detail the effect on hospitalization data including recurrent hospitalization, total number of cardiovascular/HF-related hospitalizations and duration of stay according to treatment allocation.

g) Develop a risk model for patients with HF using key baseline characteristics to allow clinicians to accurately assess prognosis in individual patients.

h) Examine the potential economic impact of treatment under a variety of circumstances and different subgroups.

i) Improve statistical methodology for IPD meta-analysis.

### Study inclusion criteria

A number of inclusion criteria were adopted to make the project methodologically sound and technically feasible. All RCTs of beta-blockers versus placebo explicitly reporting mortality as a primary or combination outcome will be included but not head-to-head comparisons with another active agent. Trials must include patients with documented symptomatic HF and only unconfounded trials will be accepted (in which one treatment group differed from another only by the beta-blocker therapy of interest). Finally, RCTs would only be included if they recruited more than 300 patients in total and planned follow-up of six months or greater. By concentrating on the larger trials (which have enrolled 95.7% of all RCT participants), the project remains practical while the amount of data missed by not including the smaller trials is minimized.

### Search strategy and eligible studies

To ensure a complete assessment of the evidence, published or unpublished RCTs were identified through computer aided searches (for example, Medline and Current Contents), scrutiny of reference lists of trials, trials registries, meeting abstracts, review articles and discussion with members of the collaborative group and with the pharmaceutical manufacturers.

The search identified 11 trials that met all inclusion criteria (with a predicted total of 18,630 participants); the Australia/New Zealand Heart Failure Study (ANZ) [10], the Beta-Blocker Evaluation Survival Trial (BEST) [11], the Carvedilol Post-Infarct Survival Control in LV Dysfunction Study (CAPRICORN) [12], the Carvedilol Hibernating Reversible Ischaemia Trial: Marker of Success Study (CHRISTMAS) [13], the Cardiac Insufficiency Bisoprolol Study (CIBIS I) [14], the Cardiac Insufficiency Bisoprolol Study II (CIBIS-II) [15], the Carvedilol Prospective Randomized Cumulative Survival Study (COPERNICUS) [16], the Metoprolol in Idiopathic Dilated Cardiomyopathy Study (MDC) [17], the Metoprolol CR/XL Randomised Intervention Trial in Congestive Heart Failure (MERIT-HF) [18], the Study of the Effects of Nebivolol Intervention on Outcomes and Rehospitalisation in Seniors with Heart Failure Study (SENIORS) [19] and the U.S. Carvedilol Heart Failure Study (US-HF) [20]. A number of large trials were discussed in detail but were not included. The Randomized Evaluation of Strategies for Left Ventricular Dysfunction (RESOLVD) Pilot Study (n = 426) was excluded as the trial had a multi-factorial design with two stages of randomization which may have confounded treatment effect [21]. Further, the beta-blocker phase of the trial involved a follow-up period of only 24 weeks. The Carvedilol ACE-Inhibitor Remodelling Mild CHF Evaluation (CARMEN) trial (n = 572) was excluded as the study design was a parallel group randomization of carvedilol plus enalapril, carvedilol plus placebo or enalapril plus placebo and examined effects on left-ventricular remodeling [22]. Additional discussions were held regarding the inclusion of CAPRICORN (the only post-infarct trial) and BEST (utilizing a pharmacologically distinct beta-blocker). The Collaborative Group decided to include these studies, but to perform sensitivity analyses for the primary outcome, as detailed in the statistical section below. Details of the included trials are presented in Table 1 and demographic variables in Table 2.

**Table 1:** Details of studies proposed for inclusion in meta-analysis

**Trial (drug) by year**	**n**	**Population**	**Inclusion criteria**	**Exclusion criteria**	**Major endpoints**	**Withdrawal/ Lost to follow-up**	**Study period**
MDC	383	Symptomatic IDC	EF <40%;	Treatment with BB or CCB; Significant CAD on angiography; Myocarditis; Life-threatening diseases; COPD requiring beta agonists; Drug or alcohol abuse; IDDM; Thyroid disease; SBP <90 mmHg; HR <45	All-cause mortality; Need for transplantation; exercise capacity; NYHA, QoL	12% metoprolol, 16% placebo /	18 months (12 after 1990); additional 3-year data
(metoprolol)			Age 16 to 75				
						one lost	
1993							
CIBIS	641	Symptomatic HF	EF <40%;	Hypertrophic/restrictive cardiomyopathy; Untreated valve disease; Awaiting bypass surgery; MI in previous three months; On heart transplantation list; IDDM; Asthma; Creatinine >300 μmol/L; Thyroid disease; Life threatening disease; SBP <100 or >160 mmHg; HR <65	All-cause mortality; Bisoprolol tolerability (based on NYHA and adverse events)	23% bisoprolol, 26% placebo /	Mean 1.9 years
						one lost	
			Age 18 to 75; NYHA III or IV				
(bisoprolol)							
1994							
US-HF	1,094	Symptomatic HF	EF ≤35%;	Major CV event/surgery within three months; Uncorrected valve disease; Myocarditis; Uncontrolled ventricular tachycardia/heart block; Clinically important hepatic or renal disease; Conditions limiting exercise or survival; Treatment with BB, CCB or class 1C antiarrhythmic agents; SBP <85 or >160 mmHg; HR <68	All-cause mortality; Hospitalization	5.7% carvedilol, 7.8% placebo /	Median 6.5 months
(carvedilol)							
						zero lost	
1996							
ANZ	415	Symptomatic HF due to CAD	EF <45%;	Coronary event/procedure within four weeks; Sick sinus, 2^nd^ or 3^rd^ degree heart block; Treadmill exercise duration <2 or >18 minutes; Myocardial or valvular disease; Treatment with BB, beta agonist or verapamil; IDDM; COPD; hepatic disease; Creatinine >250 μmol/L); Life-threatening disease; SBP <90 or >160 mmHg; HR <50	EF; Exercise duration; NYHA; Death; Hospitalization	20% carvedilol, 14%	Mean 19 months
						placebo /	
			NYHA II or III				
						zero lost	
(carvedilol)							
1997							
CIBIS II	2,647	Symptomatic HF	EF <35%;	MI/unstable angina within three months; Revascularization within six months; Prior or scheduled heart transplant; Uncontrolled 2^nd^/3^rd^ degree heart block; Creatinine >300 μmol/L; Reversible COPD; Treatment with BB, CCB or antiarrhythmic drugs other than amiodarone; SBP <100 mmHg or uncontrolled hypertension; HR <60	All-cause mortality; All-cause hospital admissions; CV mortality	15% bisoprolol, 15% placebo /	Mean 1.3 years
(bisoprolol)			NYHA III or IV				
						six lost	
1999							
MERIT-HF	3,991	Symptomatic HF	EF ≤40%;	MI/unstable angina within 28 days; BB within six weeks, CCB or amiodarone within six months; Planned or performed transplantation or implanted defibrillator; Bypass surgery or percutaneous intervention planned or in last four months; Uncorrected 2^nd^/3^rd^ degree heart block; Other serious diseases; SBP <100 mmHg; HR <68	All-cause mortality; All-cause mortality plus all-cause hospitalization	14% metoprolol, 15% placebo /	Mean 1 year
(metoprolol XL)			Age 40 to 80				
						zero lost	
1999							
COPERNICUS	2,289	Severe HF	EF <25%;	Uncorrected valvular disease or reversible cause; Prior or planned cardiac transplant; Primary pulmonary or hepatic disease; Creatinine >247.5 μmol/L; Potassium <3.5 or >5.2 mmol/L; Coronary revascularization, MI; stroke or ventricular arrhythmia within two months; Treatment with BB within two months or alpha-blocker, CCB or class I antiarrhythmic within four weeks; SBP <85 mmHg; HR <68	All-cause mortality; Hospitalization	15% carvedilol, 19% placebo /	Mean 10.4 months
(carvedilol)			NYHA III or IV				
						zero lost	
2001							
CAPRICORN	1,959	Left ventricular dysfunction post-MI	3 to 21 days post-MI;	Continued requirement of intravenous diuretics; Unstable angina; Unstable IDDM; BB indication other than HF; Inhaled beta agonists or steroids; SBP <90 mmHg or uncontrolled hypertension; HR <60	All-cause mortality; All-cause mortality or CV hospitalization; Sudden death; HF-hospitalization; Non-fatal events	20% carvedilol, 18% placebo /	Mean 1.3 years
(carvedilol)							
						endpoint-driven	
			EF ≤40%				
2001							
BEST	2,708	Symptomatic HF	EF <35%;	Reversible cause of HF or valvular disease; Untreated thyroid disease; Obstructive/hypertrophic cardiomyopathy; Pericardial disease; Amyloidosis; Myocarditis; MI within six months; Candidate for heart transplantation; Revascularization within 60 days; Unstable angina; Life expectancy <3 years; Active liver disease or excess alcohol; Creatinine >265 μmol/L; Other serious diseases; Treatment with BB within 30 days, CCB or beta-agonists within one week, class 1 antiarrhythmic within two weeks or amiodarone within eight weeks; SBP <80 mmHg; HR <50	All-cause mortality; Death from CV causes; Hospitalization; EF; Non-fatal MI; QoL	23% bucindolol, 25% placebo /	Mean 2 years
(bucindolol)			NYHA III or IV;				
						eight lost	
2001			Digoxin in all patients pre-1997				
CHRISTMAS	375	Stable HF due to CAD	EF <40%;	Women of child-bearing age; Acute CV event within three months; Hospital admission within one month; Unstable angina; Arrhythmias (for example, atrial fibrillation); Uncontrolled hypertension; COPD; Poorly controlled diabetes; Clinically relevant renal or hepatic disease; Treatment with non-dihydropiridine CCB, BB or antiarrhythmic other than amiodarone; SBP <85 mmHg; HR <60	Change in EF (hibernators vs. non-hibernators);	15% carvedilol, 7% placebo /	Mean 6.3 months
(carvedilol)			Age ≥40 years;				
						one lost	
					Regional echocardiographic contractile dysfunction;		
2003			NYHA I to III				
					Death or worsening HF.		
SENIORS	2,128	Elderly HF	Age ≥70;	Uncorrected valvular disease; Current use of BB; Significant hepatic or renal dysfunction; Stroke within three months; Pending coronary revascularization; Other serious medical conditions reducing survival; SBP <90 mmHg; HR <60	All-cause mortality or CV hospitalization; All-cause mortality; All-cause hospitalization; NYHA	27% nebivolol, 25% placebo /	Mean 21 months
(nebivolol)			HF-hospitalization within 12 months or EF ≤35% within 6 months				
						37 lost	
2005							

BB, beta-blocker; CAD, coronary artery disease; CCB, calcium channel blocker; COPD, chronic obstructive pulmonary disease; CV, cardiovascular; EF, ejection fraction; HF, heart failure; HR, heart rate; IDC, idiopathic dilated cardiomyopathy; IDDM, insulin-dependent diabetes mellitus; MI, myocardial infarction; NYHA, New York Health Association; QoL, quality of life; SBP, systolic blood pressure.

**Table 2:** Patient characteristics of studies proposed for inclusion in meta-analysis

**Trial (drug) by Year**	**n**	**Mean age**	**Male**	**Mean EF**	**Diabetes**	**On ACEi/ARB**	**Mean SBP (mmHg)**	**Mean HR (bpm)**
MDC (metoprolol) 1993	383	49	72%	22%	n/s	79%	118	91
CIBIS (bisoprolol) 1994	641	60	83%	25%	n/s	90%	126	83
US-HF (carvedilol) 1996	1,094	58	77%	23%	29%	95%	116	84
ANZ (carvedilol) 1997	415	67	80%	29%	19%	85%	n/s	n/s
CIBIS II (bisoprolol) 1999	2,647	61	80%	28%	12%	96%	130	80
MERIT-HF (metoprolol XL) 1999	3,991	64	78%	28%	25%	96%	130	83
COPERNICUS (carvedilol) 2001	2,289	63	80%	20%	26%	97%	123	83
CAPRICORN (carvedilol) 2001	1,959	63	74%	33%	22%	98%	121	77
BEST (bucindolol) 2001	2,708	60	78%	23%	36%	98%	117	82
CHRISTMAS (carvedilol) 2003	(375)*	63	90%	29%	22%	87%	126	78
SENIORS (nebivolol) 2005	2,128	76	63%	36%	26%	89%	139	79
TOTAL / WEIGHTED MEAN	18,630	63	77%	27%	23%	95%	126	81

ACEi, angiotensin converting enzyme inhibitor; ARB, angiotensin receptor blocker; EF, ejection fraction; HR, heart rate; n/s, not specified; SBP, systolic blood pressure.

* Characteristics reported are for the 305 participants with available radionuclide ventriculograms, as per the original publication.

### Primary and secondary outcomes

The primary outcome for BB-HF will be all-cause mortality, including deaths recorded after publication of the trial, where these data are available. The major secondary outcome will be the composite of all-cause mortality and cardiovascular hospitalizations. Secondary mortality outcomes include death due to acute myocardial infarction (MI), stroke, sudden cardiac death and HF-related death. Other secondary outcomes are non-fatal MI, all-cause hospitalization, cardiovascular hospitalization, HF-related hospitalization and the number and duration of hospital admissions. Drug safety outcomes will focus on discontinuation due to hypotension, bradycardia, renal impairment and HF-exacerbation.

### Statistical analysis

Due to the complexity of the statistical analyses, the following section represents the planned principal analyses; some modifications and secondary analyses are likely to emerge during the project. However, a detailed statistical analysis plan will be produced prior to the analysis.

Careful initial evaluation will be performed to ensure completeness of data, and to check consistency of the results of the primary analyses for each trial with published reports. Baseline characteristics of patients will be presented separately for each trial and overall. Continuous variables will be presented as mean and standard deviation (or median and range if not normally distributed). Binary and categorical variables will be presented as frequencies and percentages. All analyses will follow the principle of intention to treat as closely as possible. Specifically, we will include all randomized patients with outcome data.

#### Primary analysis

The primary and major secondary outcome will be analyzed using a stratified Cox regression model [23], with studies as strata. Further models will include known prognostic factors for this patient population (for example, age, gender, baseline ejection fraction, blood pressure, heart rate, diabetes and renal function). This adjusted analysis will give estimates that are more relevant to individual patients. This is a fixed effect approach and assumes that all trials are estimating a common treatment effect. Standard tests for heterogeneity will be carried out and as a sensitivity analysis a random effects approach will be followed. Hazard ratios and corresponding 95% confidence intervals will be presented, along with the corresponding *P*-value.

#### Secondary/subgroup analyses

The secondary outcomes will be analyzed in the same manner as the primary outcome.

Subgroup analyses will be used to assess the effect of beta-blockers in the following pre-specified subgroups: age, diabetes, gender, ejection fraction, atrial fibrillation and etiology of HF. The influence of age and ejection fraction on the effects of beta-blockers will be explored as continuous variables and as clinically relevant categories for other variables. A meta-analysis of interaction estimates will be used to assess whether any improvement depends on baseline LV ejection fraction, blood pressure, and the presence or absence of other concomitant cardiovascular therapies, such as ACE inhibitors, angiotensin receptor blockers, diuretics and digoxin.

Multiple imputation for missing data will be used where appropriate as a sensitivity analysis. Dichotomous outcomes will be combined using an inverse variance meta-analysis. Odds ratios and corresponding 95% confidence intervals will be presented, along with the corresponding *P*-value. To explore the influence of baseline covariates on the primary and major secondary outcomes, we will develop multivariable models using the Cox proportional hazards approach to develop a risk score. In all analyses, continuous variables will be kept continuous, and the nature of their relation to outcome evaluated using fractional polynomials.

As noted above, we also plan to perform sensitivity analyses for the exclusion (separately) of the BEST and CAPRICORN trials. If data from the eligible trials are unobtainable, then analysis using a combination of IPD and aggregate data will be considered [24].

For the economic analysis, we propose to perform a cost analysis using standard published information to provide a representative spread of health economic scenarios. Costs of care will be derived from simple drivers like hospital length of stay, medications and other treatments and a cost effectiveness analysis will be carried out based on cost per event avoided (for example, death or hospital admission). Modeling of cost effectiveness will be carried out based on specific subgroups, such as age, gender and diabetes, in a number of different healthcare models (for example, socialized care, private care and a mixed health care model), taking account of the different costs of beta-blockers. An overall population-based cost impact analysis will be derived assessing different levels of uptake of beta-blockers in heart failure with a view to estimating the cost savings of improving beta-blocker utilization.

## Discussion

Despite a wealth of information identifying the overall benefits of beta-blockers in HF for morbidity and mortality, prescription rates remain sub-optimal with consequence for both patients and healthcare providers. Current data are limited to those enrolled in the RCTs and lack sufficient statistical power to examine the harm and benefits of treatment in important patient sub-groups. For example, diabetes is present in about 25% of patients enrolled in the larger trials of beta-blockers in HF. The risk of mortality and other complications is higher in diabetics but meta-analysis of published tabular data has suggested that the absolute mortality reduction using beta-blockers may be less in patients with diabetes [25]. We will update this analysis by including data from four additional studies and uniquely, will be able to adjust for baseline covariates which may account for much of the apparent difference in effect.

Perhaps the most important patient factor that affects prescription of evidence-based therapy is age. In most population-based studies the incidence and prevalence of HF increases with age and the average age of prevalent HF is about 75 years. Most of the trials enrolled patients with a mean age of 60 to 65 years; only SENIORS recruited a population of 70 years or older. The proposed meta-analysis will allow a reliable exploration of any interaction on the effect of beta-blockers with age, albeit limited to the populations recruited in the individual studies. Similarly, most of the trials have enrolled patients with systolic dysfunction (entry ejection fraction <40%; see Table 1) and an interaction between baseline ejection fraction and the benefit of beta-blockers may exist [26].

Women with HF are under-represented in the literature and account for less than a quarter of patients in the trials listed in Table 1. Further, the benefits of beta-blockade in women are inconsistent. In the pooled CIBIS trials, all-cause mortality was similarly reduced in men and women [27] and in the US-HF study women apparently benefited more from beta-blockade (hazard ratio 0.23, 0.07 to 0.69) than men (0.41, 0.22 to 0.80) [20]. In comparison, the results from BEST, MERIT-HF and COPERNICUS found no significant benefit in women [16,28,29]. Gender also has important consequences on evaluation, treatment and prognosis in HF. We know that women with HF have different prognostic indicators than men, such as older age, more hypertension and higher ejection fraction, are less likely to receive guideline therapies, and have longer hospital stays [28,30,31].

There are also concerns that beta-blockers may worsen renal function in HF by reducing renal blood flow and glomerular filtration rate. Renal impairment is a common co-morbidity in HF that limits therapy. However, existing (under-powered) data suggest that beta-blockers are effective regardless of baseline renal function [32,33]. We aim to model the effects of beta-blockers stratified by renal function as a continuous variable, adjusting these effects for other covariates which is impossible in a simple tabular analysis. Other important controversies that can be addressed by this IPD analysis include the effects of beta-blockers in patients with HF and atrial fibrillation [34-36], and the interaction of benefit with heart rate, blood pressure changes and achieved dose [37,38]. We will also use the data to develop new insights into risk factors for death and hospitalization in HF patients. There are also important safety issues to explore, including the risk of adverse events, such as hypotension and bradycardia.

Finally, although IPD meta-analyses are considerably more resource-intensive and time-consuming than standard tabular approaches, we believe that only IPD can address the unanswered questions about the use of beta-blockers in HF. Extraction of data from published reports has significant limitations; typically outcomes are described as the number of participants that have died on each intervention over a fixed period, yielding an odds ratio. In comparison, IPD allows a full time-to-event analysis addressing each event from the time of randomization. The hazard ratio obtained provides a more appropriate view of survival and accounts for censored patients. As previously described, IPD also permits robust analyses of subgroups and the ability to estimate the interaction between covariates and treatment effect [39]. The combined sample sizes for the major variables of interest are sufficient to provide statistical power and conclusive data on the safety and efficacy of beta-blockers in HF patients.

In summary, this individual patient-data systematic review and meta-analysis will provide a definitive assessment of the role of beta-blockers in heart failure. The aims of the Collaborative Group are to reduce the burden of morbidity and mortality in heart failure patients and provide clinicians and healthcare agencies with clear guidance on which patients will benefit from beta-blocker therapy.

## Appendix A.

### A.1. Appendix: Members of BB-HF Collaborative Group

#### A.1.1. Steering Committee

Marcus Flather: Norwich Medical School, University of East Anglia, Norwich, UK.Dipak Kotecha: Clinical Trials and Evaluation Unit, Royal Brompton Hospital/Imperial College London, UK and Monash Centre of Cardiovascular Research and Education in Therapeutics, Monash University, Melbourne, VIC, Australia.Henry Krum: Monash Centre of Cardiovascular Research and Education in Therapeutics, Monash University, Melbourne, VIC, Australia.Luis Manzano: Department of Medicine, Universidad de Alcala, Hospital Universitario Ramon y Cajal, Madrid, Spain.

#### A.1.2. Statistical Team

Nicola Williams: Centre for Statistics in Medicine, University of Oxford, Oxford, UK.Douglas Altman: Centre for Statistics in Medicine, University of Oxford, Oxford, UK.

#### A.1.3. Collaborative Group

Bert Andersson: Department of Cardiology, Sahlgrenska University Hospital, Göteborg, Sweden.John Cleland: University of Hull, Kingston upon Hull, UK.Andrew Coats: University of East Anglia, Norwich, UK.Mike Domanski: National Heart, Lung and Blood Institute, Bethesda, MD, USA.Guliz Erdem: Clinical Trials & Evaluation Unit, Royal Brompton Hospital/Imperial College London, UK.Marilena Grana: A. Menarini Farmaceutica Internazionale, Firenze, Italy.Per Haglund: AstraZeneca Clinical Information Science, Mölndal, Sweden.Åke Hjalmarson: Sahlgrenska University Hospital; Göteborg, Sweden.Philippe Lechat: Agence Française de Sécurité Sanitaire des Produits de Sante, Saint Denis, France.Alain Leizorovicz: Service de Pharmacologie Clinique, Université de Lyon, Lyon Cedex, France.Mary Ann Lukas: GlaxoSmithKline Cardiovascular and Metabolism Medicine Development Center, Philadelphia, PA, USA.Wilfried Meyer: Merck Serono Global Medical Affairs, Darmstadt, Germany.Milton Packer: UT Southwestern Medical Center, Dallas, TX, USA.Alan Rigby: Academic Cardiology, Castle Hill Hospital, Kingston upon Hull, UK.Giuseppe Rosano: Department of Medical Sciences, IRCCS San Raffaele Pisana, Roma, Italy.Michael Roughton: Clinical Trials and Evaluation Unit, Royal Brompton Hospital/Imperial College London, London, UK.Rosemary Schroyer: GlaxoSmithKline Cardiovascular and Metabolism Medicine Development Center, Philadelphia, PA, USA.Marcelo Shibata: Division of Cardiology, University of Alberta, Edmonton, AB, Canada.Hans Wedel: Nordic School of Public Health, Göteborg, Sweden.John Wikstrand: Wallenberg Laboratory, Göteborg University, Göteborg, Sweden.Thomas von Lueder: Monash Centre of Cardiovascular Research and Education in Therapeutics, Monash University, Melbourne, VIC, Australia.and in memoriam; Philip Poole-Wilson: National Heart and Lung Institute, Imperial College London, London, UK.

## Abbreviations

ACE: Angiotensin converting enzyme; ANZ: Australia/New Zealand Heart Failure Study; ARB: Angiotensin receptor blockers; BB-HF: Beta-Blockers in Heart Failure Collaborative Group; BEST: Beta-Blocker Evaluation Survival Trial; CAPRICORN: Carvedilol Post-Infarct Survival Control in LV Dysfunction Study; CHRISTMAS: Carvedilol Hibernating Reversible Ischaemia Trial: Marker of Success Study; CIBIS I: Cardiac Insufficiency Bisoprolol Study; CIBIS-II: Cardiac Insufficiency Bisoprolol Study II; COPERNICUS: Carvedilol Prospective Randomized Cumulative Survival Study; HF: Heart failure; IPD: Individual Patient Data; LV: Left Ventricular; MDC: Metoprolol in Idiopathic Dilated Cardiomyopathy Study; MERIT-HF: Metoprolol CR/XL Randomised Intervention Trial in Congestive Heart Failure; MI: Myocardial infarction; NYHA: New York Heart Association; RCTs: randomized controlled trials; SENIORS: Study of the Effects of Nebivolol Intervention on Outcomes and Rehospitalisation in Seniors with Heart Failure Study; US-HF: U.S. Carvedilol Heart Failure Study.

## Competing interests

The majority of the collaborative group have received speaker fees, honoraria or grant support from pharmaceutical companies involved in beta-blocker therapies.

The BEST trial was principally sponsored by the US National Heart, Lung and Blood Institute and the Department of Veterans Affairs Cooperative Studies Program. All other trials were funded by pharmaceutical companies.

## Authors’ contributions

DK participated in the design of the study, manages the collaborative group and performs data management and statistical analysis. LM, GE, HK and MDF participated in the design and coordination of the study. DGA and NW participated in the design of the study and the statistical analysis. All authors drafted, read and approved the final manuscript.

## Supplementary Material

Additional file 1 Please see attached file for BB-HF data request. Click here for file
