# Supplementary material for: Individual patient data meta-analysis of beta-blockers in heart failure: rationale and design
Source: Syst Rev. 2013 Jan 18;2:7. doi: 10.1186/2046-4053-2-7 (PMC3564787; doi:10.1186/2046-4053-2-7)
Supplement: Additional file 1 — Please see attached file for BB-HF data request. [file 2046-4053-2-7-S1.doc]

**DATA REQUEST v3.3; 26 Dec 2010**

# STUDY-LEVEL DATA

Study Name (Abbreviation):

Total number randomized:

Number in treatment arm:

Number in placebo arm:

Date first patient randomized:  DAY MONTH YEAR

Date last patient randomized: DAY MONTH YEAR

Date of final follow-up: DAY MONTH YEAR

Unit of measurement – Height:       Not applicable

Unit of measurement – Weight:       Not applicable

Unit of measurement – Creatinine:       Not applicable

Unit of measurement – Sodium:       Not applicable

Unit of measurement – Uric acid:       Not applicable

Unit of measurement – Haemoglobin:       Not applicable

Does the study have quality of life measurements? Yes  No

If yes, name of QoL tool:

# INDIVIDUAL PATIENT DATA

Explanatory notes:

Variable number. Variable Description [*variable name*] coding [or string variable details]

## A. PATIENT IDENTIFIER & RANDOMIZATION DATA

1. Study [*study*] 1 MDC

2 CIBIS I

3 US Carvedilol

4 ANZ

5 CIBIS II

6 MERIT-HF

7 COPERNICUS

8 CAPRICORN

9 BEST

10 SENIORS

11 CHRISTMAS

1. Patient identifier [*pid*] [STUDY CODE, string var (20 char max)]
2. Patient initials [*patinit*] [string var (3 char max)]
3. Date of randomization [*date*] D D / M M / Y Y Y Y
4. Treatment allocation [*alloc*] 1 Placebo

2 Beta-blocker

## B. BASELINE DATA (recorded prior or at randomization)

1. Date of birth [*dob*] D D / M M / Y Y Y Y
2. Gender [*gender*] 1 Male

2 Female

9 Data unavailable

1. Race [*race*] 1 White/Caucasian

2 African/African-American

3 Asian

4 Other

9 Data unavailable

1. Race specify [*raceother*] [string var]
2. Aetiology of heart failure [*cause*] 1 Ischaemic heart disease

2 Idiopathic dilated cardiomyopathy

3 Other / Unknown

9 Data unavailable

1. Year of heart failure diagnosis [*hfdiag*] Y Y Y Y
2. History of myocardial infarction [*mi*] 0 No

1 Yes

9 Unknown / Unavailable

1. Date of last myocardial infarction [*midate*] D D / M M / Y Y Y Y
2. History of angina pectoris [*angina*] 0 No

1 Yes

9 Unknown / Unavailable

1. History of percutaneous intervention [*pci*] 0 No

1 Yes

9 Unknown / Unavailable

1. History of coronary bypass surgery [*cabg*] 0 No

1 Yes

9 Unknown / Unavailable

1. History of stroke [*stroke*] 0 No

1 Yes

9 Unknown / Unavailable

1. History of peripheral arterial disease [*pad*] 0 No

1 Yes

9 Unknown / Unavailable

1. History of hypertension [*ht*] 0 No

1 Yes

9 Unknown / Unavailable

1. Diabetes mellitus [*dm*] 0 No

1 Yes

9 Unknown / Unavailable

1. Classification of diabetes [*dmtype*] 1 Insulin-dependent

2 Non insulin-dependent

9 Unknown / Unavailable

1. History of atrial fibrillation / flutter [*histaff*] 0 No

1 Yes

9 Unknown / Unavailable

1. History of chronic airways disease [*copd*] 0 No

1 Yes

9 Unknown / Unavailable

1. Smoking [*smoke*] 1 Current smoker

2 Ex-smoker

3 Never smoked

9 Unknown / Unavailable

1. Height [*height*]
2. Weight [*weight*]
3. Systolic blood pressure [*sbp*] [mmHg – sitting value preferred]
4. Diastolic blood pressure [*dbp*] [mmHg – sitting value preferred]
5. Resting heart rate [*hr*] [beats per minute]
6. NYHA class [*nyha*] 1 NYHA Class I

2 NYHA Class II

3 NYHA Class III

4 NYHA Class IV

9 Unknown / Unavailable

1. Creatinine level [*creat*]
2. Sodium value *[sodium]*
3. Uric acid *[uric]*
4. Haemoglobin level [*hb*]
5. Ejection fraction at entry [*lvef*] [as fraction, 0._ _ ]
6. ECG abnormal [*ecg*] 0 No

1 Yes

9 Unknown / Unavailable

1. ECG rhythm [*ecgrhyt*] 0 Sinus rhythm

1 Atrial fibrillation/flutter

2 Heart block

3 Paced

9 Unknown / Unavailable

1. Bundle branch block [ecgbbb] 0 No bundle branch block

1 Left bundle branch block

2 Right bundle branch block

3 Unspecified

9 Unknown / Unavailable

1. ECG signs of ischaemia [*ecgisch*] 0 No

1 Yes [Q-waves, ST/T changes]

9 Unknown / Unavailable

1. Left-ventricular hypertrophy on ECG [*ecglvh*] 0 No

1 Yes [as per study definition]

9 Unknown / Unavailable

1. Proteinuria on urinalysis [*prot*] 0 No

1 Yes [1+ on dipstick; ≥30mg/dL]

9 Unknown / Unavailable

1. 6-minute walk distance [base*6mwd*] distance walked [metres]
2. Minnesota Quality of Life [baseqol] total score

## C. BASELINE DRUG TREATMENT (at randomization/study entry)

1. Aspirin [*aspirin*] 0 No

1 Yes

9 Unknown / Unavailable

1. Other antiplatelet agent [*antiplat*] 0 No

1 Yes

9 Unknown / Unavailable

1. Oral anticoagulant [*anticoag*] 0 No

1 Yes

9 Unknown / Unavailable

1. Loop diuretic [*diurloop*] 0 No

1 Yes [Furosemide, Bumetanide, Ethacrynic acid, Torsemide]

9 Unknown / Unavailable

1. Dose of furosemide [*loopdose*] [mg]
2. Thiazide diuretic [*diurthia*] 0 No

1 Yes [Bendrofluazide, Bendroflumethiazide, Benzthiazide, Chlorothiazide, Chlorthalidone, Cyclopenthiazide, Hydrochlorothiazide, Indapamide, Metolazone, Xipamide]

9 Unknown / Unavailable

1. Other potassium-sparing diuretic [*diurksp*] 0 No

1 Yes [Amiloride, Triamterene – N.B. Does not include Spironolactone, see 54]

9 Unknown / Unavailable

1. Cardiac glycoside [*digoxin*] 0 No

1 Yes

9 Unknown / Unavailable

1. ACE inhibitor [*acei*] 0 No

1 Yes [Captopril, Enalapril, Ramipril, Quinapril, Perindopril, Lisinopril, Benazepril, Fosinopril, Trandalopril]

9 Unknown / Unavailable

1. Angiotensin receptor blocker [*arb*] 0 No

1 Yes [Candesartan, Eprosartan, Irbesartan, Telmisartan, Valsartan, Losartan]

9 Unknown / Unavailable

1. Aldosterone antagonist [*aldo*] 0 No

1 Yes [Spironolactone, Eplerenone, Canrenone]

9 Unknown / Unavailable

1. Vasodilator [*vaso*] 0 No

1 Yes [Hydralazine, long-acting nitrates]

9 Unknown / Unavailable

1. Amiodarone [*amio*] 0 No

1 Yes

9 Unknown / Unavailable

1. Other anti-arrhythmic [*arryth*] 0 No

1 Yes

9 Unknown / Unavailable

1. Calcium channel blocker [*ccb*] 0 No

1 Yes

9 Unknown / Unavailable

1. Statin [*statin*] 0 No

1 Yes [Atorvastatin, Cerivastatin, Fluvastatin, Lovastatin, Pitavastatin, Pravastatin, Rosuvastatin, Simvastatin]

9 Unknown / Unavailable

1. Bronchodilator therapy [*brochodil*] 0 No

1 Yes

9 Unknown / Unavailable

## D. ADVERSE CLINICAL EVENTS

1. Death during study period [*death*] 0 No

1 Yes

1. Date of death [*deathdate*] D D / M M / Y Y Y Y
2. Cause of death [*deathcause*] 1 Acute myocardial infarction

2 Sudden death

3 Heart failure

4 Other cardiac

5 Stroke

6 Other vascular/thrombo-embolic

7 Non-cardiovascular

8 Unknown

[1-4, cardiac; 1-6, cardiovascular]

1. Death after study completed [*deathpost*] 0 No

1 Yes

1. Date of death after study completed [*deathdatepost*] D D / M M / Y Y Y Y
2. Cause of death after study completed 1 Acute myocardial infarction

[*deathcausepost*] 2 Sudden death

3 Heart failure

4 Other cardiac

5 Stroke

6 Other vascular/thrombo-embolic

7 Non-cardiovascular

8 Unknown

[1-4, cardiac; 1-6, cardiovascular]

1. Number of all-cause hospital admissions [*hospall*]
2. Number of cardiovascular admissions [*hospcv*]
3. Number of HF-related hospital admissions [*hosphf*]
4. Admission date, 1^st^ CV admission [*hosp1adm*] D D / M M / Y Y Y Y

1. Discharge date, 1^st^ CV admission [*hosp1dis*] D D / M M / Y Y Y Y
2. Type of 1^st^ cardiovascular admission [*hosp1hf*] 0 Not heart failure related

1 Heart failure related

9 Unknown / Unavailable

1. Admission date, 2^nd^ CV admission [*hosp2adm*] D D / M M / Y Y Y Y
2. Discharge date, 2^nd^ CV admission [*hosp2dis*] D D / M M / Y Y Y Y
3. Type of 2^nd^ cardiovascular admission [*hosp2hf*] 0 Not heart failure related

1 Heart failure related

9 Unknown / Unavailable

1. Admission date, 3^rd^ CV admission [*hosp3adm*] D D / M M / Y Y Y Y
2. Discharge date, 3^rd^ CV admission [*hosp3dis*] D D / M M / Y Y Y Y
3. Type of 3^rd^ cardiovascular admission [*hosp3hf*] 0 Not heart failure related

1 Heart failure related

9 Unknown / Unavailable

1. Admission date, 4^th^ CV admission [*hosp4adm*] D D / M M / Y Y Y Y
2. Discharge date, 4^th^ CV admission [*hosp4dis*] D D / M M / Y Y Y Y
3. Type of 4^th^ cardiovascular admission [*hosp4hf*] 0 Not heart failure-related

1 Heart failure-related

9 Unknown / Unavailable

1. Admission date, 5^th^ CV admission [*hosp5adm*] D D / M M / Y Y Y Y
2. Discharge date, 5^th^ CV admission [*hosp5dis*] D D / M M / Y Y Y Y
3. Type of 5^th^ cardiovascular admission [*hosp5hf*] 0 Not heart failure-related

1 Heart failure-related

9 Unknown / Unavailable

1. Non-fatal myocardial infarction [*aemi*] 0 No

1 Yes

1. Date of first myocardial infarction [*aemi1*] D D / M M / Y Y Y Y
2. Date of second myocardial infarction [*aemi2*] D D / M M / Y Y Y Y
3. Non-fatal stroke [*aestroke*] 0 No

1 Yes

1. Date of first stroke [*aestroke1*] D D / M M / Y Y Y Y
2. Date of second stroke [*aestroke2*] D D / M M / Y Y Y Y
3. *New* atrial fibrillation during study period [*newaf*] 0 No

1 Yes

9 Unknown / Unavailable

1. *New* ischaemic ECG changes during study 0 No

[*newecgisch*] 1 Yes

9 Unknown / Unavailable

1. Study drug/placebo discontinued due to 0 No

adverse event [*disc*] 1 Yes

1. Discontinuation due to hypotension [*dischypo*] 0 No

1 Yes

1. Discontinuation due to bradycardia [*discbrad*] 0 No

1 Yes

1. Discontinuation due to impaired renal function 0 No

[*discren*] 1 Yes

1. Discontinuation due to heart failure exacerbation 0 No

[*dischf*] 1 Yes

1. Discontinuation due to respiratory function 0 No

[*discresp*] 1 Yes

1. Discontinuation due to other reason [*discother*] 0 No

1 Yes – complete next field

1. Discontinuation reason [*disctext*] [free text]
2. Date study drug/placebo discontinued for any D D / M M / Y Y Y Y

reason [*discdate*]

## E. INTERIM FOLLOW-UP (preferably a mid-point visit)

1. Date of follow-up visit [*smdate*] D D / M M / Y Y Y Y
2. Treatment dose achieved [*smdose*] [mg]
3. Systolic blood pressure [*smsbp*] [mmHg – sitting value preferred]
4. Diastolic blood pressure [*smdbp*] [mmHg – sitting value preferred]
5. Resting heart rate [*smhr*] [beats per minute]
6. Creatinine level [*smcreat*]
7. Ejection fraction [*smlvef*] [as fraction, 0._ _ ]
8. NYHA Class [*smnyha*] 1 NYHA Class I

2 NYHA Class II

3 NYHA Class III

4 NYHA Class IV

9 Unknown / Unavailable

1. Weight [*smweight*]
2. 6-minute walk distance [sm*6mwd*] distance walked [metres]
3. Minnesota Quality of Life [smqol] total score
4. Aspirin [*smaspirin*] 0 No

1 Yes

9 Unknown / Unavailable

1. Other antiplatelet agent [*smantiplat*] 0 No

1 Yes

9 Unknown / Unavailable

1. Oral anticoagulant [*smanticoag*] 0 No

1 Yes

9 Unknown / Unavailable

1. Loop diuretic [*smdiurloop*] 0 No

1 Yes [Furosemide, Bumetanide, Ethacrynic acid, Torsemide]

9 Unknown / Unavailable

1. Dose of furosemide [*smloopdose*] [mg]
2. Thiazide diuretic [*smdiurthia*] 0 No

1 Yes [Bendrofluazide, Bendroflumethiazide, Benzthiazide, Chlorothiazide, Chlorthalidone, Cyclopenthiazide, Hydrochlorothiazide, Indapamide, Metolazone, Xipamide]

9 Unknown / Unavailable

1. Other potassium-sparing diuretic [*smdiurksp*] 0 No

1 Yes [Amiloride, Triamterene – N.B. Does not include Spironolactone, see 123]

9 Unknown / Unavailable

1. Cardiac glycoside [*smdigoxin*] 0 No

1 Yes

9 Unknown / Unavailable

1. ACE inhibitor [*smacei*] 0 No

1 Yes [Captopril, Enalapril, Ramipril, Quinapril, Perindopril, Lisinopril, Benazepril, Fosinopril, Trandalopril]

9 Unknown / Unavailable

1. Angiotensin receptor blocker [*smarb*] 0 No

1 Yes [Candesartan, Eprosartan, Irbesartan, Telmisartan, Valsartan, Losartan]

9 Unknown / Unavailable

1. Aldosterone antagonist [*smaldo*] 0 No

1 Yes [Spironolactone, Eplerenone, Canrenone]

9 Unknown / Unavailable

1. Vasodilator [*smvaso*] 0 No

1 Yes [Hydralazine, long-acting nitrates]

9 Unknown / Unavailable

1. Amiodarone [*smamio*] 0 No

1 Yes

9 Unknown / Unavailable

1. Other anti-arrhythmic [*smarryth*] 0 No

1 Yes

9 Unknown / Unavailable

1. Calcium channel blocker [*smccb*] 0 No

1 Yes

9 Unknown / Unavailable

1. Statin [*smstatin*] 0 No

1 Yes [Atorvastatin, Cerivastatin, Fluvastatin, Lovastatin, Pitavastatin, Pravastatin, Rosuvastatin, Simvastatin]

9 Unknown / Unavailable

1. Bronchodilator therapy [*smbrochodil*] 0 No

1 Yes

9 Unknown / Unavailable

## F. FINAL FOLLOW-UP

1. Lost to follow-up [*fnlost*] 0 No

1 Yes

1. Last date of follow-up [*fndate*] D D / M M / Y Y Y Y
2. Treatment dose achieved [*fndose*] [mg]
3. Systolic blood pressure [*fnsbp*] [mmHg – sitting value preferred]
4. Diastolic blood pressure [*fndbp*] [mmHg – sitting value preferred]
5. Resting heart rate [*fnhr*] [beats per minute]
6. Creatinine level [*fncreat*]
7. Haemoglobin level [*fnhb*]
8. Ejection fraction [*fnlvef*] [as fraction, 0._ _ ]
9. NYHA Class [*fnnyha*] 1 NYHA Class I

2 NYHA Class II

3 NYHA Class III

4 NYHA Class IV

9 Unknown / Unavailable

1. Weight [*fnweight*]
2. 6-minute walk distance [fn*6mwd*] distance walked [metres]
3. Minnesota Quality of Life [fnqol] total score
4. Aspirin [*fnaspirin*] 0 No

1 Yes

9 Unknown / Unavailable

1. Other antiplatelet agent [*fnantiplat*] 0 No

1 Yes

9 Unknown / Unavailable

1. Oral anticoagulant [*fnanticoag*] 0 No

1 Yes

9 Unknown / Unavailable

1. Loop diuretic [*fndiurloop*] 0 No

1 Yes [Furosemide, Bumetanide, Ethacrynic acid, Torsemide]

9 Unknown / Unavailable

1. Dose of furosemide [*fnloopdose*] [mg]
2. Thiazide diuretic [*fndiurthia*] 0 No

1 Yes [Bendrofluazide, Bendroflumethiazide, Benzthiazide, Chlorothiazide, Chlorthalidone, Cyclopenthiazide, Hydrochlorothiazide, Indapamide, Metolazone, Xipamide]

9 Unknown / Unavailable

1. Other potassium-sparing diuretic [*fndiurksp*] 0 No

1 Yes [Amiloride, Triamterene – N.B. Does not include Spironolactone, see 153]

9 Unknown / Unavailable

1. Cardiac glycoside [*fndigoxin*] 0 No

1 Yes

9 Unknown / Unavailable

1. ACE inhibitor [*fnacei*] 0 No

1 Yes [Captopril, Enalapril, Ramipril, Quinapril, Perindopril, Lisinopril, Benazepril, Fosinopril, Trandalopril]

9 Unknown / Unavailable

1. Angiotensin receptor blocker [*fnarb*] 0 No

1 Yes [Candesartan, Eprosartan, Irbesartan, Telmisartan, Valsartan, Losartan]

9 Unknown / Unavailable

1. Aldosterone antagonist [*fnaldo*] 0 No

1 Yes [Spironolactone, Eplerenone, Canrenone]

9 Unknown / Unavailable

1. Vasodilator [*fnvaso*] 0 No

1 Yes [Hydralazine, long-acting nitrates]

9 Unknown / Unavailable

1. Amiodarone [*fnamio*] 0 No

1 Yes

9 Unknown / Unavailable

1. Other anti-arrhythmic [*fnarryth*] 0 No

1 Yes

9 Unknown / Unavailable

1. Calcium channel blocker [*fnccb*] 0 No

1 Yes

9 Unknown / Unavailable

1. Statin [*fnstatin*] 0 No

1 Yes [Atorvastatin, Cerivastatin, Fluvastatin, Lovastatin, Pitavastatin, Pravastatin, Rosuvastatin, Simvastatin]

9 Unknown / Unavailable

1. Bronchodilator therapy [*fnbrochodil*] 0 No

1 Yes

9 Unknown / Unavailable
